# Address before the I. H. A.

**Published:** 1887-07

**Authors:** J. T. Kent

**Affiliations:** President


### ADDRESS BEFORE THE INTERNATIONAL HAHNEMANNIAN ASSOCIATION AT ITS EIGHTH ANNUAL MEETING,

By Professor J. T. Kent, M. D., President.

Fellow-members of the International Hahnemannian Association: It is with pleasure that I welcome you to your eighth annual meeting; to one which promises to exceed in interest and profit even our last session.

In the past, this Association has accomplished some very useful work for the cause it espouses. Let us hope it will do even more in the future! And what *is* the cause we espouse; or, in other terms, why this Association?

It was certainly for no idle purpose, nor for any senseless caprice, that our oldest and most respected members left the American Institute and formed this separate Association; it is equally true that we of the junior profession did not join this Association for any selfish or useless purpose. Was not this Association formed solely for the purpose, as expressed, of perpetuating and developing true Homœopathy? Was it not felt at the time of its organization that the hour had come for true men to arouse themselves and work for the science they loved? Had they not heard all the principles which Hahnemann had taught, and which the experience of many had proven to be true, villified and abused; had not, in short, all true Homœopathy been driven from the Institute? The homœopathic school, then as now, was divided into two parties—the one representing eclectic methods and practice, the other the principles and practice of Hahnemann, of Gross, of Bœnninghausen, of Hering. The time had come when all practitioners had to decide which of the parties they should assist. And let it be to the eternal glory of these men that they chose rather to be right than to be with the majority!

In the history of the American Institute, we may read a warning for us. In its first years the Institute was composed of able and true men, and its purpose was for truth and usefulness. But little by little eclectics were allowed to creep into its membership, and soon, behold! the whole body is eclectic. Let us then beware whom we elect members, let our censors be even over-scrupulous lest a wolf creep in in sheep's clothing. Let no member sign any application for membership unless he knows the physician personally and is *very* sure he is qualified to serve with us. Too great caution cannot be observed in this matter. It is not great numbers that we want, but men of truth and purpose.

While much caution may be judiciously exercised in this matter of electing new members, let us not repel those who, though not yet with us, are in sympathy with our purpose, and whose presence would be welcome. Let us not therefore erect any Chinese wall of exclusion, but merely exercise all proper precaution to prevent evil. Let no good man be excluded by personal malice; nor any useless man elected to serve personal ambition. As well stated in the preface to our last volume of transactions:

"Personal interests or ambitions have no place here, but only *what* is truth."

Without doubt all will assent to this assertion, but many will inquire, and most rightly, too, *What is truth?* This question has been asked many, many times, and of all subjects. In this case, limiting our statement to what is true in therapeutics, we unhesitatingly assert the law of similars to be true; to be a proven *fact*. Has it not been found operative in all diseases and in all countries? can fuller demonstration be needed?

"*It is true; let it stand*," we all exclaim.

It may be well to remark that while our law is a fixed fact, we must never forget that our school is not to be stationary. The law is complete and perfect; our knowledge of the extent of its usefulness is very incomplete and imperfect. The law is fixed, the school is progressive.

Eclectics, building upon the uncertain sands of *theory*, need to be continually rebuilding, as each new theory causes a shifting of their foundation. Homœopathists, building upon the unchangeable rock of *law*, need never rebuild.

Our foundation then being firm, we need only develop and improve the superstructure. Our knowledge of the extent and usefulness of the law of similars has increased since Hahnemann's day; let us see to it that we continue to improve, and always in the right way.

The law, being of divine origin, is complete, perfect, and fixed; the school, being composed of erring humanity, is incomplete, imperfect, and changeable.

While many willingly concede this much to the homœopathic law, they yet desire something more; they would like to have *liberty*, license, "to use their best judgment;" to be free to treat anomalous cases by non-homœopathic measures if, in their judgment, such may at any time be needed.

There is growing up such a tendency to the so-called scientific that our young men stand in danger of being drawn into this vortex of confusion. This scientific vortex looks wonderful; it is so strong! What can there be in the science of medicine but a knowledge of how to cure the sick? The scientific physician, when asked what he knows, must say: *I know how to cure the sick.* If he really knows this he has knowledge and is scientific. If he has not this knowledge, which he pretends to possess, he is a pretender and a fraud.

What is there of value in this word "scientific," when all the pretenders in medicine make use of it? These, most of all, cry "We are the scientific." "We teach science." The amount of science depends entirely on how much the instructor possesses, for "a stream cannot rise higher than its source."

The "eclectics" claim to teach the most scientific (?) of all, because they select the good from all schools of medicine. Who has guided them to this great wisdom? Do they pretend to have a law or a philosophy to enable them to select the wheat and leave the chaff? No. Such a thing does not belong to their pretensions. They even claim the greatest empiricism to be the highest order of science. The greater the chaos and confusion the greater the science.

The cry of the unbelieving does not strengthen their scientific position when their only appeal is to the microscope and to common sense. *Common sense is opposed at all times to cultivated intelligence.* The man of lowest intelligence can prove that he must have a dose that can be seen and handled to cure him of his aches, by appealing to common sense. The mongrel makes use of the same reason and argument to condemn us that the allopathist resorts to to convict the mongrel—appeal to common sense and belief.

Ten men may stand and affirm each, "I did not see," and one man states "I did see," and who of the eleven would the meanest court in the land accept as competent to give evidence? The one knows what the ten do not know.

The ten declare they have tried the high potencies and have failed to secure curative results. What have they demonstrated? *Nothing but their own ignorance* of the manner of using these potencies. But they say they cure with the low. I do not believe they cure with the low, because of the best reasoning. It is logical to suppose or presume that a physician who can cure with the high, can cure with the low, but the demonstration is entirely wanting to show that the physician can cure *with the low* and cannot cure with the high. Men who know how to select a remedy have confidence in that remedy and go on gaining yearly in this knowledge; men who are ignorant of the powers of the selected remedy of course have not gained the confidence necessary to cure with it, and they mix other means and other medicines.

It has been recently stated in a medical journal that there are logical reasons for deserting Homœopathy for allopathy; that is, for abandoning law for empiricism. The idea is fallacious, and no sensible reason has ever been adduced in its support. There can be only one excuse for this change—and that is *failure!* And this failure has never yet been shown to be due to any insufficiency of the homœopathic law, but is always easily traced to the incapacity of him who uses it. All are liable to err. Let him who thinks he cannot sin cast the first stone at our law.

Concerning the oft-made plea for liberty of medical opinion and action, we would remark that no one is free from the obligations of law; the greater your work, the higher you advance, just by so much do you rivet the chains of responsibility. Only the beggar in the gutter is free to do as he will. No one can grant a physician success in practice whose practice does not of itself secure success.

If one practice Homœopathy he will secure homœopathic success; if he practice allopathy, he will gain only the meagre results of allopathy. No resolutions of learned bodies can change this rule. We are freemen; free to do and to practice as we please; but our success will be measured by our practice, and our title as homœopaths or eclectics be given accordingly as we practice the one or the other, and we all know the greatest measure of success is attained by a strict adherence to the law of similars, the minimum dose, and the single remedy. The Homœopathy of Hahnemann gives the greatest success, the greatest freedom, and the greatest honor. No man can practice empiricism and honestly claim to be a homœopath; such are "living a lie," as an allopath has asserted. The eclectic is a slave, bound by error; the homœopath is free, emancipated by truth. A great poet declares, "He is a freeman whom truth makes free, and all are slaves beside."

Let not this Association harbor or indorse in any way, even by absence of rebuke, any form of false teaching. Let it be distinctly understood that we do fully and honestly believe, collectively and individually, the resolutions of this Association, as adopted. We have declared that these resolutions "completely and fully represent the therapeutic opinion and practice" of this Association. Let it be shown to the outside world that we mean what we have said. We do most assuredly believe Hahnemann's *Organon of the Healing Art* to be the only true guide in therapeutics. Let us not, then, tolerate any teaching which seeks to pervert or abridge this master work in any way. We have asserted, as our belief, that the only true guide for a prescription is the totality of the symptoms and the proven drug. Let us not, then, prescribe upon any other basis; it cannot be homœopathic nor wise to do so. We cannot allow to be true any teaching which seeks to controvert this fundamental principle of homœopathic practice. He who recommends the building of therapeutics upon any new theory or upon any other basis than that prescribed by this law, is no homœopath and has no fellowship in this Association. Successful practice cannot be based upon pathological theories. Whether these theories teach one to prescribe for a pathological condition or for a presumed dyscrasia, it matters not; both are un-homœopathic and both are unsuccessful.

The adoption of drug proving by Hahnemann, first introduced two great features into medicine, and these are *certainty* and *prevision*. We are sure a drug will cure in the sick such symptoms as it has produced upon the healthy; we are enabled by this certainty to *predict*, before the trial of a drug, what it will cure. For these grand features of its art, medicine is indebted to Samuel Hahnemann—see to it that no fault of ours destroys his noble work. In short, it is to be remembered that the basis of a homœopathic prescription is the symptom of the patient, the question of the dose is secondary. The size of the dose can never make the remedy homœopathic to the case.

In this matter of dose, some err upon one side and some upon the other. So we see that while some believe an imperfectly selected drug may be made to do the work of the perfect simillimum if it be "pushed" or exhibited in crude doses; on the other hand, we find some who are disposed to assent to almost any prescription so it be given high enough. Both these parties are in error. While we cannot dogmatize upon this question of dose, all here will agree that the better the selection, *i. e.*, the nearer we come to the perfect simillimum, the less medicine we need give. This proposition may be stated again in other words. It is the experience of our best prescribers that the simillimum will cure most cases best if given high and in one dose, or at least a few doses. Indeed, experience tells us that the high potencies are always the best; this is experience, however, and not law. But the converse of this proposition is *not true*, that a badly selected drug may be made to do good work by giving much of it. This idea is the cause of most of the mongrelism of the day.

In published reports of clinical cases, we find evidence of the necessity for careful examination of the patient. Hahnemann laid the greatest stress upon this examination, telling us how to do it, and saying, in effect, that a patient well examined was half cured. Unless this careful examination be made, one cannot get all those peculiar, characteristic symptoms which Hahnemann has declared must be the deciding symptoms. All cases have many symptoms, which are to be found under many drugs, and are hence of little value in deciding our choice of a remedy. Each case should have, and probably does have, some peculiar symptoms; these we are to get. These we *must* get; and our examination of a patient is incomplete so long as we possess only a list of common and general symptoms. It should be our task to question and examine the patient until such peculiar symptoms are found. We hear much complaint of the insufficiency of our *Materia Medica*, of the uselessness of our repertories, but most generally the failure to prescribe correctly and even easily is not due to the want of good books, but to this lack of careful and thoughtful examination of the patient. Forget not this, that the greatest cures the world has ever witnessed have been made by the earlier homœopaths with a much less complete library than we now possess. After selecting the proper remedy, we must not forget that it is of prime importance to give it in proper dose, and not to change too soon nor to repeat too frequently. Never change a remedy unless the changed symptoms call for another; never repeat the dose (or change remedy) when the patient is improving. For a fuller and a better understanding of the true healing art, you are to study and to restudy the *Organon*. Our purpose in these few remarks has not been to teach this art, but merely to call attention to a few salient points; to give admonition upon a few prominent features which cannot be too steadily kept in view.

This Association, it has been said, was organized for an especial purpose, and that purpose was to promulgate and develop Homœopathy. In pursuance of this work, the purifying and completing of the *Materia Medica* must be our chief concern. It is the foundation of our art. Our *Materia Medica* once corrupted and perverted, clinical success becomes impossible. We may again take warning by the fate of the American Institute, for it, too, started forty odd years ago, to do this same work; and for some years the Institute did good service in this study. But as it grew eclectic, the Institute became enamored of the false siren named *progressive science*, and all truth was abandoned. Let us beware lest a like fate overtake this Association.

The *Materia Medica* is to be developed by careful and thorough provings of new drugs; we repeat, careful and thorough provings, for most of the modern provings are worthless, having been carelessly and improperly made. One is afraid to prescribe upon them; afraid to trust valuable lives to such careless work. How differently do we feel when we prescribe one of the old, reliable remedies. Then security begets quiet reliance and success crowns our efforts.

At our last meeting, a good beginning was made in this study of the *Materia Medica*, and your Bureau gives promise of great usefulness and interest for this meeting. In all of our work we must strive to emulate the energy and zeal of Hahnemann and of his early disciples; they were indeed masters. Nowhere does one's knowledge of therapeutics and medical ability show forth to better advantage than in this of proving drugs and revising the *Materia Medica*. To do it well the best talent and the greatest zeal are required; but this need not deter us from the work, for ability and zeal are easily to be found in our ranks.

The *Materia Medica* is to be enriched by clinical observations, and here also we may again take pattern by Hahnemann's careful work. The admission of clinical symptoms into our *Materia Medica* must be done with the greatest caution. They can only be incorporated after the most searching inquiry, and then should always be so marked that we can tell the clinical from the pathogenetic. The hasty and inconsiderate adoption of clinical symptoms is certainly an evil; and if pursued to any great extent will render the *Materia Medica* unreliable. Every practitioner is not a reliable judge of the value of a clinical confirmation. Even reliable clinical confirmations need only be noted when peculiar or characteristic; of common, general symptoms we have an abundance.

The clinical symptom is only admissible to fill up the gaps left by imperfect provings, or in cases where provings cannot be obtained. Though some of the best symptoms now in use are of clinical origin, as a general rule they cannot be considered as certain and reliable as the pathogenetic.

Besides the proving of drugs and the careful, conscientious noting of clinical symptoms, we can also do a useful work in marking clinical verifications of pathogenetic symptoms. A symptom produced upon a healthy person and cured in a sick person becomes doubly reliable. There can be no doubt about the value of such symptoms.

The most dangerous manner of perpetuating homœopathic truth is to mix it with uncertainty or mystery. There are some things about the art of nealing that pertain to the scientific, of which not one is more important than the *proven drug*. A member may state that he has cured somebody with an unproved drug, and he may fail to demonstrate the homœopathicity of the so-called cure, because of the lack of evidence that can only be obtained from the provings. There are many good things so involved in mystery that the time is not ripe to discuss them. The relations of Homœopathy to them must be first demonstrated or this organization cannot recognize them. The allopathist reports cures on unsupported opinion, and we reject these because he has no demonstration. If this same allopathist reports a cure of vomiting by *Ipecac*, the homœopathist can accept it as a real cure, because it is what can be expected. Experiment as you may on the healthy with new medicines, the sick man demands a remedy for his sickness the likeness of which has been found in a pathogenesis.

In no way can we perpetuate pure philosophy but by adhering to the proven drug in all our discussions. Better rule out all the fragmentary guesswork and make every report show its relation between drug and disease in the manner designated in our philosophy. The Publication Committee should reject, without fear or favor, all papers with reports of cures where we have not had access to the record of provings. Of what value is the cure without the proving? Save the cures until you have given us the proving.

By thorough and careful work we will some day complete a *Materia Medica* whose every symptom will have been repeatedly verified. Then, indeed, will our art become the exact science predicted for it. Such is the end for which we labor. A great stride toward such an end will be made when we have in completed form the *Guiding Symptoms*, by the late Dr. Hering. These are now promised, and if given us as that master mind left them (not as some lesser minds may think they should be given), our school will secure a treasure. A very opposite of this great work of Hering's is the so-called *Encyclopœdia of Drug Pathogensy*, which seems to be a confused mass of mangled provings. We have more than once attempted to gather assistance from its garbled and condensed pages, but have always been baffled. That it has any value we are unable to see. It is to be hoped it has a purpose, as much labor seems to have been spent upon it, and much expected of it.

There is another point to which your attention may be profitably directed, and that is to secure greater care in selecting our medicines and more care in manufacturing our potencies. It seems as though carelessness were also creeping into our pharmaceutics. The greatest discretion must be exercised in selecting proper material for our pharmacopœia and in their preparation. The same preparation, especially in the use of our vegetable remedies, should be used in the prescribing as was used in the proving. We do not mean the same potency, but the same pharmaceutical preparation. Impure or uncertain drugs will, of course, not correspond in their effects upon the sick to the action of a purer drug used in the proving. The physician and the prover should use the same preparation. Without doubt, many of our failures may be justly laid to some imperfection in our drug preparations.

During the past year little worthy of note has occurred in the medical world. In the old school new theories have arisen and old ones have died. This is the old, old story with these scientists! Among ourselves the work seems to be steadily progressing for the better. The successful meeting held a year ago at Saratoga has been productive of much good, has shown the outside world that this is a *working* association of genuine homœopaths. Such successful meetings cannot fail to have a beneficial effect upon the homœopathic school.

And now we meet for the eighth time to greet each other, and to work for the perpetuation of the art of healing known as Homœopathy. We have come together from the remote quarters of the land to sharpen a common faith by another year of busy experience. This organization has been separated from the masses of all grades in medicine, a mere handful, that has been called a respectable minority, and it can even now see the gulf that yawns behind it. With independence we are to go on climbing the mountain of homœopathic *truth*. Some say we are at the top. Be not so sure; we have but climbed a foothill; soon will we see a mountain beyond, with but the faintest trace of human footprints. We follow on, though the mountain side be steep and thorny, led by the light of truth. Soon the toilers grow weary and their number becomes smaller. In the distant past there is a multitude, while the valleys below still throng with conflicting millions. The few toil on up the steep and rocky mountain side, steeper, more rocky as they press onward. The distance brings to view the heavens, dotted with nebulous sky and space beyond. There is to be seen another mountain far away, and much higher, which is yet to be climbed, upon which, through the clear sky, above the clouds, behold the immortal Hahnemann.

